# Elizabethkingia meningoseptica Causing Infection in a Chronic Kidney Disease Patient With Hepatitis B Positive Status: Unraveling the Hidden Culprit

**DOI:** 10.7759/cureus.56254

**Published:** 2024-03-16

**Authors:** Gautam N Bedi, Sourya Acharya, Smruti A Mapari, Pranjal Kashiv, Sushrut Gupta

**Affiliations:** 1 Medicine, Jawaharlal Nehru Medical College, Datta Meghe Institute of Higher Education & Research, Wardha, IND; 2 Obstetrics and Gynecology, Jawaharlal Nehru Medical College, Datta Meghe Institute of Higher Education & Research, Wardha, IND; 3 Nephrology, Jawaharlal Nehru Medical College, Datta Meghe Institute of Higher Education & Research, Wardha, IND

**Keywords:** hepatitis b, hemodialysis, end-stage renal disease, chronic kidney disease, elizabethkingia meningoseptica

## Abstract

Elizabethkingia meningoseptica is a rare gram-negative bacterium recognized for its propensity to induce hospital-acquired infections, particularly in individuals with compromised immune systems and those equipped with indwelling medical devices. Its notorious resistance to a broad spectrum of antibiotics poses a considerable challenge in treatment protocols, contributing to its emergence as a significant cause of heightened mortality rates among critically ill patients. Herein, we present a case of E. meningoseptica infection in a patient afflicted with end-stage renal disease (ESRD) undergoing maintenance hemodialysis, concurrently grappling with ESRD, and a positive status for hepatitis B. This case report aims to shed light on the intricate complexities involved in diagnosing and managing such infections within this intricate clinical context.

## Introduction

Elizabethkingia meningoseptica, initially characterized by Elizabeth O. King in 1959, is a non-motile, non-fermentative, oxidase-positive gram-negative bacillus [1]. While widely distributed in nature, it was not traditionally associated with human colonization [2]. However, it has been found to inhabit various environmental niches within healthcare settings, including sink basins, taps, and ventilator circuits, posing a potential source of infection in hospital environments. This bacterium has garnered attention for causing hospital-acquired infections (HAIs) such as pneumonia, meningitis, and sepsis, particularly among immunocompromised individuals [2].

One of the notable challenges in managing infections caused by E. meningoseptica is its resistance to many commonly used broad-spectrum antibiotics, including beta-lactams, carbapenems, aminoglycosides, and colistin. This resistance profile complicates treatment regimens, often necessitating alternative approaches [3]. Additionally, the limited availability of antimicrobial susceptibility data further exacerbates the difficulty in selecting appropriate therapeutic interventions for infections caused by this pathogen.

In the context of this case report, we present a scenario wherein E. meningoseptica infection occurred in a patient concurrently diagnosed with chronic kidney disease (CKD), end-stage renal disease (ESRD) undergoing maintenance hemodialysis, and hepatitis B infection. This constellation of underlying medical conditions presents a complex clinical scenario, further compounded by the challenges posed by the intrinsic resistance of E. meningoseptica to commonly prescribed antibiotics. The case underscores the importance of recognizing and addressing the unique challenges in diagnosing and managing infections caused by E. meningoseptica, particularly in patients with multiple comorbidities such as CKD, ESRD, and viral hepatitis.

## Case presentation

**Figure 1:**
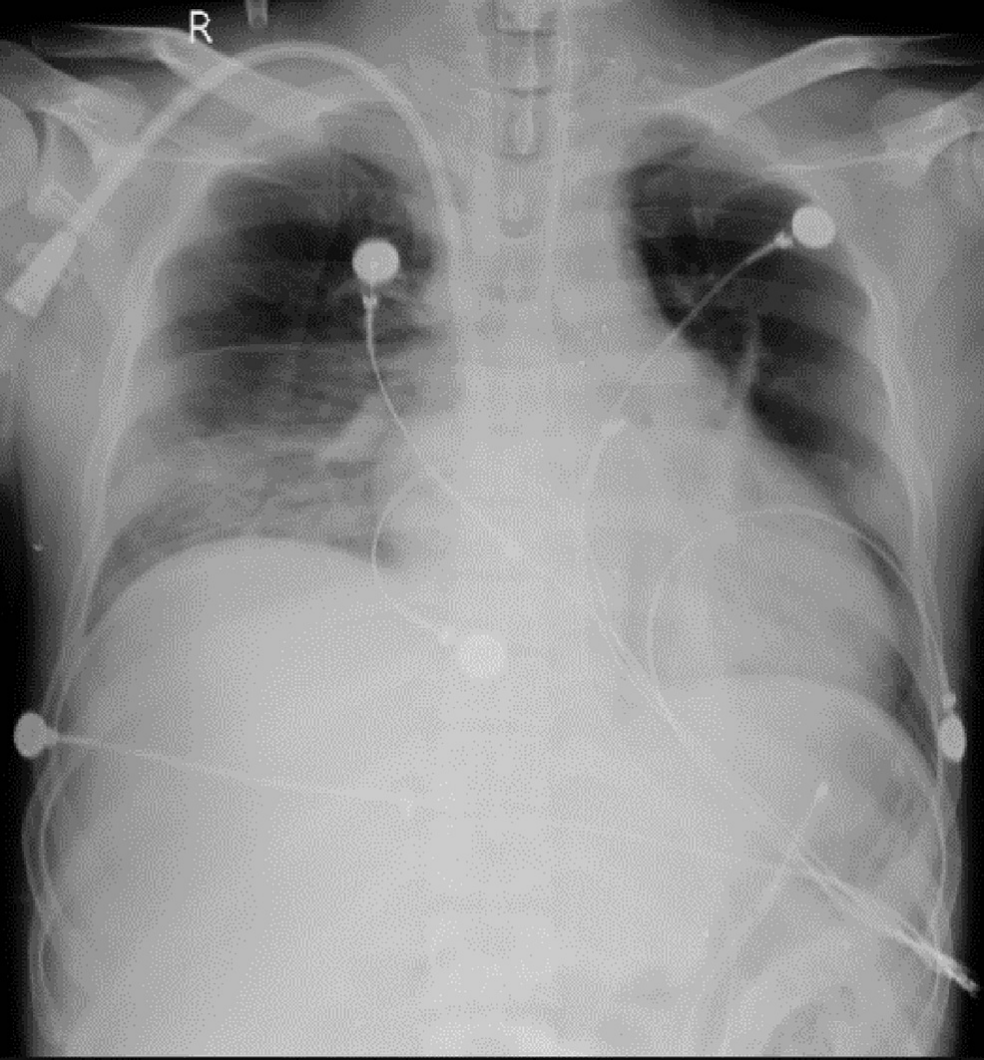
Chest X-ray showing pulmonary edema

A 28-year-old male presented to the emergency department complaining of shortness of breath and fever persisting for three days. He had a history of stage 4 CKD, diagnosed three years prior due to hypertensive nephropathy, and had been undergoing maintenance hemodialysis for the past nine months. Additionally, he tested positive for hepatitis B surface antigen (HBsAg) and had undergone multiple blood transfusions. He denied experiencing chest pain, palpitations, headaches, loose stools, nausea, or vomiting. Upon examination, his pulse rate was 66 beats per minute, blood pressure was 130/80 mmHg, respiratory rate was 24 breaths per minute, oxygen saturation was 85% on room air, and temperature was 38.5°C. Lung auscultation revealed equal bilateral air entry Figure 1. The patient exhibited pallor and bilateral pedal edema. With a Glasgow Coma Scale score of 5 and an inability to maintain adequate oxygen saturation on room air, he was transferred to the intensive care unit (ICU). His oxygen saturation improved to 96% in the ICU via a face mask with 10 liters of oxygen. Emergency intubation and insertion of Foley and Ryles tubes were performed under strict aseptic precautions.

Laboratory investigations revealed leukocytosis (white blood cell count of 15,500/mm³), elevated C-reactive protein, and abnormal liver function tests. Despite round-the-clock treatment with antipyretics, the patient continued to experience fever spikes. His symptoms persisted, accompanied by increasing total leukocyte counts, anemia, thrombocytopenia, and a raised procalcitonin level of 12 ng/mL (normal <0.15), suggestive of sepsis. Two blood cultures and a urine sample for culture were drawn from different sites. Laboratory investigations are described in Table 1.

**Table 1:** Summary of progression of laboratory parameters

Day	Investigations						
	Hemoglobin	Total Leukocyte Count (TLC)	Platelet	Urea	Creatinine	Serum Sodium	Serum Potassium
Reference Range	13-15 gm%	4000-11000 mm^3^	1.5 to 4.5 L/cumm	9-20 mg/dl	0.6-1.2 mg/dl	137-145 mmol/liter	3.5 to 5.1 mmol/liter
Day 1	3.4	15500	2.07	244	24.3	146	5.2
Day 2	5.2	11000	1.56	58	8.2	140	3.6
Day 4	7	5200	0.92	57	7.7	148	3.3
Day 5	7.2	6800	0.8	85	8.6	146	3.4
Day 9	6.8	9700	1.35	86	8.1	137	3.7
Day 12	7.4	7800	2.63	56	8.9	139	4.6

The patient was initiated on intravenous (IV) meropenem 1 gram stat followed by 500 mg every 12 hours, IV clindamycin 600 mg every 12 hours, IV antipyretics, antiviral medication (tab entecavir 0.5 mg once a week), antihypertensive therapy, and nebulization with asthalin. Blood culture results indicated the growth of a gram-negative bacillus, subsequently identified as *Elizabethkingia meningoseptica* via matrix-assisted laser desorption/ionization time-of-flight (MALDI-TOF) mass spectrometry. The isolate was found to be susceptible only to levofloxacin and cefoperazone based on sensitivity testing. Consequently, IV levofloxacin 750 mg once daily was promptly initiated. The patient exhibited a remarkable response to treatment, with improved vital signs and reduced total leukocyte counts to normal levels by the third day following the initiation of levofloxacin.

**Figure 2:**
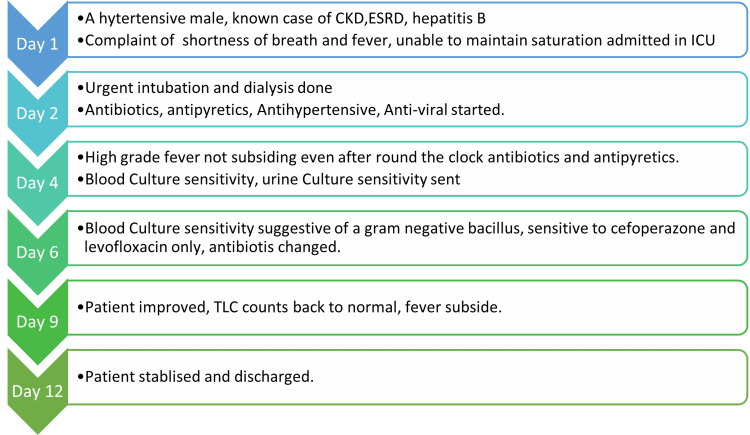
Clinical course of the patient in the hospital The corresponding author created this image. CKD: Chronic kidney disease, ESRD: End-stage renal disease, TLC: Total leukocyte count

After 72 hours, the blood culture results were sterile. The patient's clinical course throughout the hospitalization is depicted in Figure 2. This encounter marked the first experience with this rare pathogen in the ICU, prompting stringent adherence to aseptic practices and institutional protocols to control and prevent the further spread of infection. Ultimately, the patient was transferred to the general ward and discharged from the hospital in stable condition.

**Figure 3:**
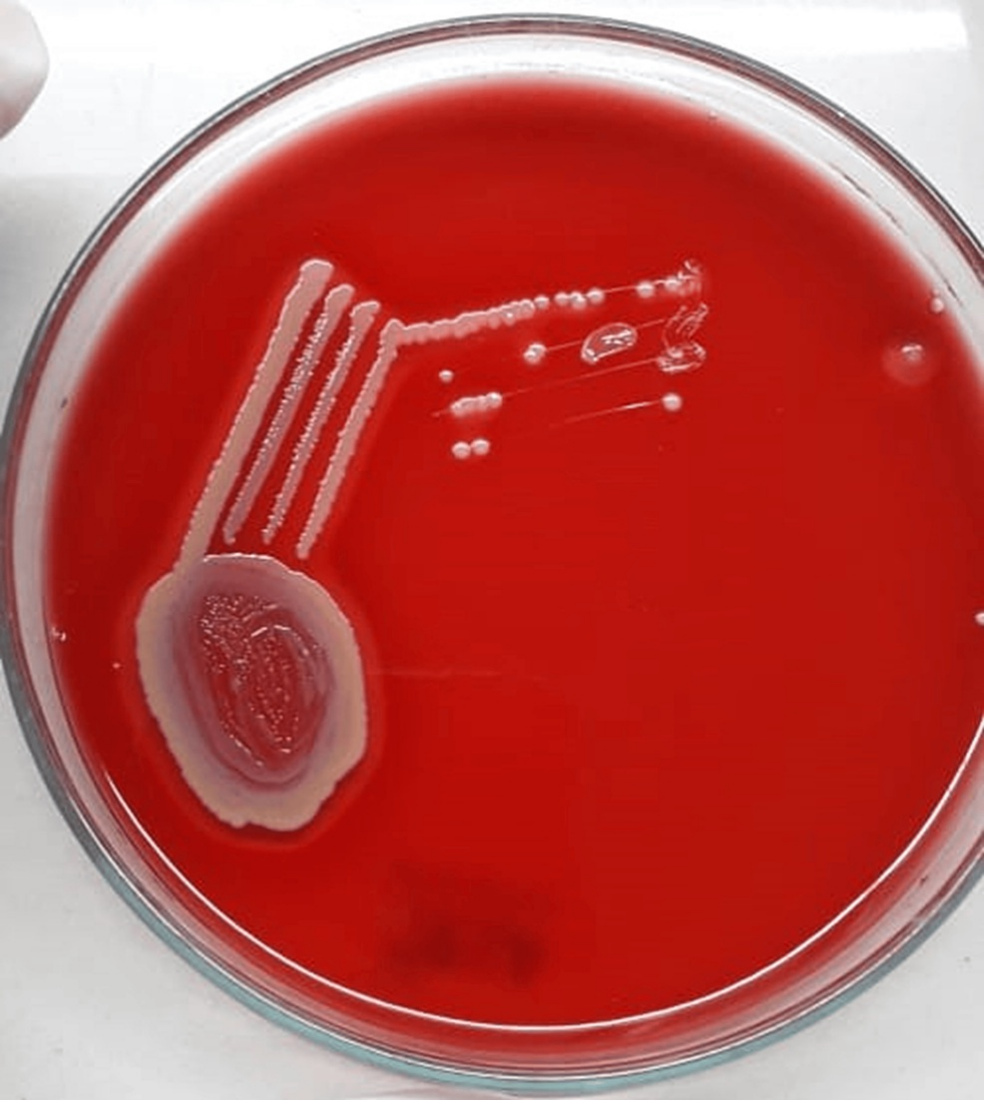
Blood agar showing wet raised colonies with clear margins suggestive of Elizabethkingia meningoseptica

**Figure 4:**
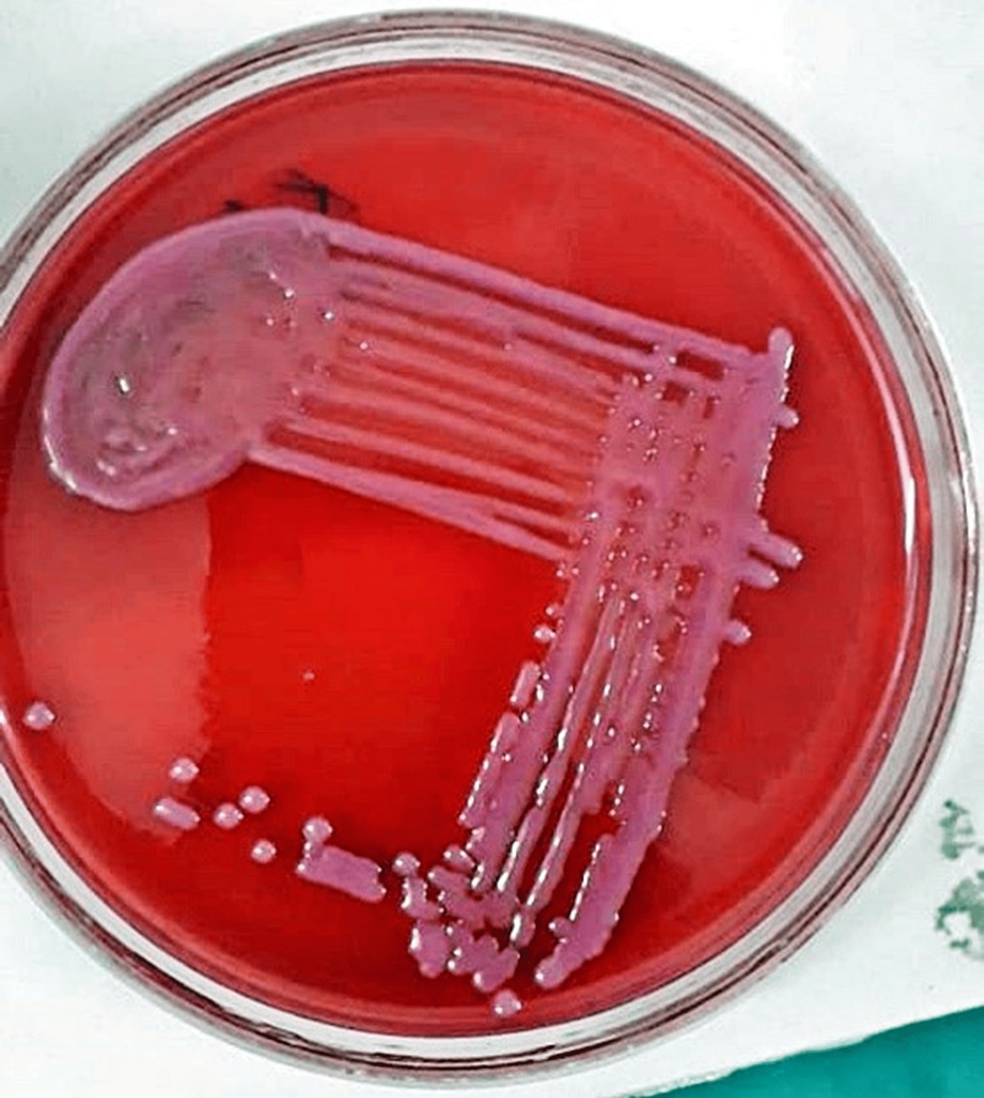
MacConkey agar suggestive of Elizabethkingia meningoseptica

Figures 3, 4 illustrate blood agar and MacConkey agar cultures, respectively, displaying characteristics indicative of *E. meningoseptica.*

## Discussion

E. meningoseptica is a gram-negative, rod-shaped bacterium widely distributed in nature and recognized as an opportunistic pathogen responsible for HAIs [4]. Over the past decade, there has been a noticeable increase in the incidence of E. meningoseptica infections. Risk factors associated with these infections include prolonged hospital stays, prior use of high-end broad-spectrum antibiotics, immunosuppression, underlying medical conditions, and indwelling medical devices. The mortality rate for infections caused by this organism is high due to the lack of effective therapeutic regimens and its intrinsic resistance to antibiotics commonly used to manage infections caused by gram-negative bacteria. Paradoxically, E. meningoseptica is susceptible to antibiotics typically used to treat gram-positive bacterial infections, such as rifampicin, ciprofloxacin, vancomycin, and trimethoprim-sulfamethoxazole [5].

Although E. meningoseptica infections in immunocompromised hosts have been recognized, clinical data regarding these infections remain limited, especially in cases originating from India. Khan et al. documented that previous exposure to high-end broad-spectrum gram-negative antibiotics, such as carbapenems and colistin, predisposes individuals to nosocomial infections caused by opportunistic pathogens like Elizabethkingia spp. [6]. In a case series study by Govindaswamy et al., all patients with E. meningoseptica infections had a recent history of hospitalizations and were on mechanical ventilation in the ICU, with a reported high mortality rate of 75% [7].

In our case, a young hypertensive patient with ESRD developed sepsis due to E. meningoseptica. The organism was isolated 10 days after admission to the hospital. The patient had multiple hospital admissions over the past three years and a history of prior exposure to antibiotics, details of which were unavailable. It is speculated that previous usage of broad-spectrum antibiotics before the current admission may have contributed to the development of this infection [8]. The patient's immunocompromised status, presence of an indwelling device (hemodialysis catheter), multiple antimicrobial exposures in the past, and prolonged ICU stay were identified as risk factors for sepsis caused by this rarely reported pathogen. In a previous multicenter study from Greece, E. meningoseptica was identified as the second most common cause of gram-negative infections in a dialysis unit [9].

The isolation of E. meningoseptica from paired blood cultures and the patient's rapid response to antimicrobial therapy tailored according to susceptibility testing provide conclusive evidence of the etiological role of this organism. While previous cases reported in the literature have shown poor prognosis [10], our patient fully recovered and was discharged. E. meningoseptica exhibits unusual resistance patterns and mechanisms, underscoring the importance of prompt diagnosis, sensitivity testing, and stringent infection control measures to prevent outbreaks. In this case, the patient's immunocompromised state, as evidenced by CKD, ESRD, and hepatitis B infection, likely contributed to susceptibility to this opportunistic pathogen.

## Conclusions

E. meningoseptica emerges as a significant pathogen, particularly among immunocompromised individuals with indwelling medical devices. Its resistance to commonly prescribed antibiotics for gram-negative bacterial infections poses a formidable challenge for healthcare providers. This case underscores the importance of considering uncommon pathogens in immunocompromised patients, particularly those with comorbidities like CKD and hepatitis B. Early detection and appropriate management, including utilizing antimicrobial susceptibility testing to tailor therapy, play a pivotal role in achieving successful outcomes in such complex cases. Further research is imperative to enhance our understanding of the epidemiology and optimal treatment strategies for E. meningoseptica infections in this patient population.
